# Prognostic significance of epidermal growth factor receptor in locally advanced esophageal squamous cell carcinoma for patients receiving chemoradiotherapy

**DOI:** 10.3892/ol.2014.1881

**Published:** 2014-02-13

**Authors:** ZHENHUA GAO, XUE MENG, DIANBIN MU, XINDONG SUN, JINMING YU

**Affiliations:** 1 Department of Radiation Oncology, Shandong Cancer Hospital and Institute, University of Jinan, Jinan, Shandong 250117, P.R. China; 2 Department of Pathology, Shandong Cancer Hospital and Institute, University of Jinan, Jinan, Shandong 250117, P.R. China

**Keywords:** concurrent chemoradiotherapy, immunohistochemistry, prognostic marker, esophageal squamous cell carcinoma, epidermal growth factor receptor

## Abstract

The aim of the current study was to investigate the prognostic significance of epidermal growth factor receptor (EGFR) in patients with locally advanced esophageal squamous cell carcinoma (ESCC) receiving concurrent chemoradiotherapy (CCRT). In total, 47 patients with locally advanced ESCC who were treated with CCRT were included in the present study. The chemotherapeutics comprised of 5-fluorouracil (750–1,000 mg/m^2^/day; days one to five) and cisplatin (30 mg/m^2^/day; days one to three) in combination with radiation therapy (~60 Gy), which was performed as the initial treatment. EGFR expression was compared with the clinicopathological features, local recurrence, metastasis status and overall survival (OS). Overall, EGFR overexpression (percentage of immunoreactive tumor cells, ≥50%) was identified in 59.6% of the patients. The median survival time (MST) of the EGFR-positive group was 15 months and the MST of the EGFR-negative group was 23.5 months. A significant correlation was observed between EGFR overexpression and poor OS (P=0.024). EGFR overexpression was found to exhibit a correlation with lymph node metastasis (P=0.011), but no correlation was identified with other clinicopathological features. In addition, a correlation was identified between OS and gender (P=0.021), age (P=0.018), depth of invasion stage (P=0.035) and tumor location (P=0.023). EGFR overexpression determined by pretreatment biopsy may be a clinically useful biomarker for predicting the OS of ESCC patients.

## Introduction

Esophageal carcinoma is one of the most common types of malignancy in China, and squamous cell carcinoma (SCC) is the main histological type. Concurrent chemoradiotherapy (CCRT) is an accepted standard treatment for patients with locally advanced esophageal squamous cell carcinoma (ESCC). However, prognosis for these patients remains poor (1–3), with a five-year overall survival (OS) rate of ~20% (4). The median survival time (MST) of all patients with T3–4M0 esophageal cancer who received CCRT was demonstrated to be only 16 months (5). Local recurrence and distant metastasis following definitive chemoradiation are the primary patterns of failure (6). Therefore, predicting the failure patterns and OS following CCRT is important. However, identification of reliable markers predicting treatment outcome following CCRT remains limited in the previous literature (7). In the surgical groups of previous studies, epidermal growth factor receptor (EGFR) overexpression has been found in ESCC and may predict the postoperative recurrence and OS (6,8–10). The clinical importance of EGFR overexpression remains unsettled in ESCC patients undergoing CCRT (11). In the present study, the prognostic relevance of EGFR was studied in locally advanced ESCC.

## Materials and methods

### Patients

In total, 47 locally advanced ESCC patients with a median age of 63 years (range, 45–72 years) were admitted to the Shandong Cancer Hospital and Institute, University of Jinan (Jinan, China). All patients fulfilled the following criteria: i) histologically confirmed ESCC; ii) no previous treatment; iii) endoscopically evaluable primary lesions; iv) Karnofsky Performance Status scale of 70–100; v) retained function of the major organs (bone marrow, heart, liver and kidneys); vi) no significant medical disease (such as myocardial infarction and pneumonectasis); vii) clinically diagnosed T2–4, N_any_ and M_any_ (Union for International Cancer Control, 6th edition; 2002); viii) physical examination and computed tomography (CT) performed prior to and following treatment; ix) received 5-fluorouracil and cisplatin (CF scheme); and x) written informed consent obtained prior to treatment. All patients were administered the same regimen of CCRT.

### Immunohistochemical staining methods

Histological analysis confirmed that all esophageal tumors were squamous cell carcinoma. All pretherapeutic endoscopic biopsy specimens were examined for EGFR expression. Immunohistochemical staining was performed with labeled streptavidin biotin (LSAB) method using a Dako LSAB kit (Dako, Carpinteria, CA, USA). Primary antibody used for the immunohistochemical staining was anti-EGFR monoclonal antibody (dilution of 1:60; clone 31G7; Cytomed GmbH, Baden-Baden, Germany).

Formalin-fixed, paraffin-embedded biopsy samples were cut into 4 μm sections. Following deparaffinization, the sections were incubated three times in a microwave oven for 10 min and incubated with 0.3% H_2_O_2_ for 30 min. Next, these sections were incubated with the primary antibody. Following six washes in phosphate-buffered saline, sections were incubated with rabbit monoclonal antibodies against EGFR for 20 min at room temperature. The primary antibodies were localized by the sequential application of biotinylated goat polyclonal anti-rabbit IgG gout immunoglobulins and streptavidin-peroxide conjugate (Dako). Immunostaining was visualized by developing the slides in diaminobenzidine (Dako) and counterstaining with Mayer’s hematoxylin (Abcam, Cambridge, UK). Finally, the slides underwent alcohol and xylene immersion and were mounted for examination. For the negative controls, the primary antibody solutions were replaced with blocking buffer.

### Staining evaluation

The sections were evaluated by two pathologists who were not informed of the results of chemoradiotherapy and the patients’ follow-up. The immunoreactivity of EGFR was characterized into the following five grades according to the percentage of immunoreactive tumor cells: 0, 0–4% positive tumor cells; 1, 5–24%; 2, 25–49%; 3, 50–74%; and 4, 75–100%. Staining grades of 3 and 4 were defined as positive for EGFR expression, while staining grades of 0, 1 and 2 were defined as negative, consistent with previous interpretations of EGFR in ESCC (12).

### Treatment schedule

Chemotherapy and radiotherapy were initiated on the same day. Patients received a total radiation dose of ~60 Gy, administered in 30 fractions (1.8–2 Gy per fraction; five times per week). Radiation was delivered by high-energy (≥6 MV) linear accelerators as a requirement. Three-dimensional treatment planning was used to ensure adequate dose delivery to the target while simultaneously limiting the total dose to normal structures. All fields were treated each day (five times per week). The gross tumor volume was defined as any evidence of disease as documented by pretreatment staging procedures, including CT, positron emission tomography or endoscopic ultrasonography. The clinical target volume was defined as the gross tumor volume plus inclusion of the regional draining lymphatics based on the primary tumor location. The planning target volume was based on tumor size as assessed by CT or endoscopy (whichever was larger), with superior and inferior borders extending 5 cm beyond the tumor and lateral borders extending 1.5 cm beyond the tumor. A barium swallow radiograph was also obtained at the time of treatment simulation to confirm the location of the tumor and esophagus. The spinal cord dose did not exceed 45.0 Gy. Doses to normal lung tissue were calculated by dose-volume histograms. The maximum dose to the entire heart was limited to 40.0 Gy, but a dose as high as 45.0 Gy could be administered to <50% of the heart. Chemotherapeutics consisted of the protracted infusion of 5-fluorouracil (750–1,000 mg/m^2^/day) on days one to five in combination with cisplatin (30 mg/m^2^/day) with adequate hydration and antiemetics continuous intravenous drip coverage between days one and three A total of two cycles of chemotherapeutics were performed during radiotherapy at four-week intervals. This was followed by two more periods of chemotherapeutics with the same doses performed at three-week intervals, three weeks following the completion of radiotherapy (13).

### Follow-up and observational indices

Patients were followed up at regular intervals (every three to six months) after CCRT. The follow-up included CT and barium swallow radiograph. Endoscopic ultrasonography was adopted when abnormal esophagus was found by the abovementioned examinations. In addition, the time of local recurrence, distant metastasis and OS were documented. OS was defined as the interval between the date of CCRT initiation and the date of mortality or final follow-up. The deadline of the follow-up was December 20, 2012.

### Statistical analysis

The SPSS software package, version 13.0 (SPSS, Inc., Chicago, IL, USA) was used for statistical analysis. A logistic regression analysis was applied to evaluate the association between the expression of EGFR and clinicopathological features. Survival curves of the patients were calculated by the Kaplan-Meier method and analyzed by the log-rank test. The prognostic significance of clinicopathological factors was assessed using the Cox proportional-hazards regression model. Two-sided significance levels of P<0.05 were considered to indicate a statistically significant difference.

## Results

### Patient characteristics

All patients with locally advanced ESCC treated with CCRT at the Shandong Cancer Hospital and Institute, Jinan University (Jinan, China) between December 2008 and November 2011 were candidates for the present study. In total, 47 patients with a median age of 63 years (range, 45–72 years) fulfilled the inclusion criteria and their clinicopathological features are presented in Table I. All patients belonged to TNM stage II/III.

### Immunoreactivity

**Figure 1:**
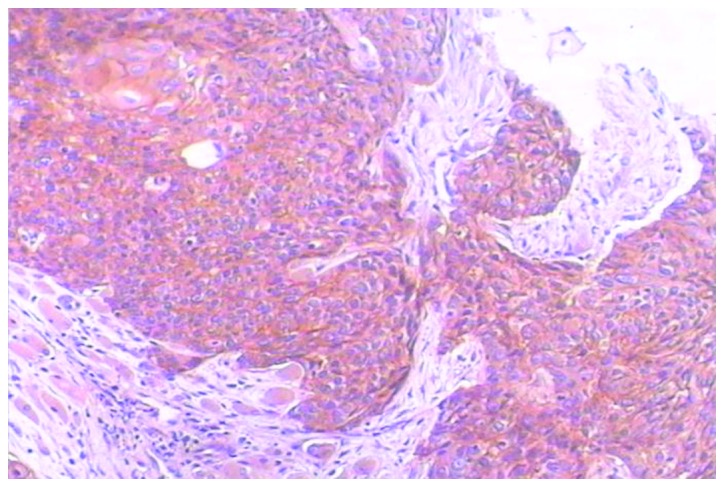
Representative immunohistochemical EGFR staining of a biopsy specimen prior to concurrent chemoradiotherapy. EGFR-positive; the percentage of immunoreactive tumor cells with staining grades of three or four are:50–74% and 75–100%, respectively. Magnification, ×100. EGFR, epidermal growth factor receptor.

**Figure 2:**
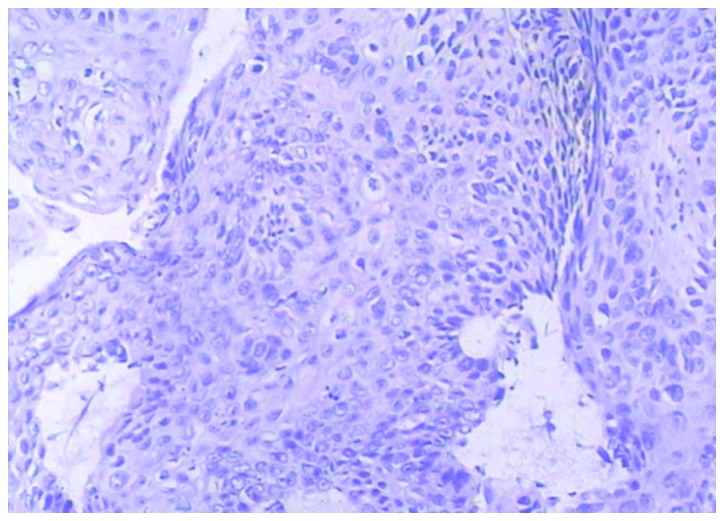
Representative immunohistochemical EGFR staining of a biopsy specimen prior to concurrent chemoradiotherapy. EGFR-negative; the percentage of immunoreactive tumor cells with staining grades of 0, 1 and 2 are: 0–4%, 5–24% and 25–49%, respectively. Magnification, ×100. EGFR, epidermal growth factor receptor.

All 47 samples were immunohistochemically detected for EGFR. EGFR expression was observed in the cell membrane and cytoplasm. Positive expression of EGFR (Fig. 1) in ESCC cells was observed in 28 (59.6%) cases; grade 3 in 11 (23.4%) cases and grade 4 in 17 (36.2%) cases. In total, 19 (40.4%) cases were EGFR-negative (Fig. 2); grade 0 in two (4.2%) cases, grade 1 in seven (14.9%) cases and grade 2 in 10 (21.3%) cases. EGFR overexpression was found to correlate with the presence of lymph node metastasis (P=0.011; Table II). By contrast, no correlation was detectable between EGFR overexpression and gender (P=0.120), age (P=0.882), tumor differentiation grade (P=0.582), tumor location (P=0.314), depth of invasion (T; P=0.593) and distant metastasis (M; P=0.051).

### Survival analyses

**Figure 3:**
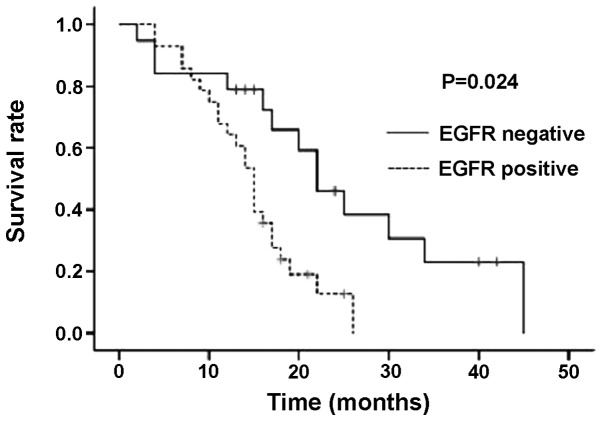
Survival curves for 47 esophageal squamous cell carcinoma patients who received concurrent chemoradiotherapy, according to EGFR expression. A significant difference in overall survival was identified between the positive and negative EGFR expression groups. EGFR, epidermal growth factor receptor.

The median duration of follow-up was 15 months and only three patients were lost to follow-up. A difference in OS was identified between patients with and without EGFR overexpression. Fig. 3 shows the survival curves according to EGFR expression using Kaplan-Meier analysis. The MST of all 47 patients in the present study who received CCRT was 16.5 months. The MST of the EGFR-positive group was 15 months and the MST of the EGFR-negative group was 23.5 months. A significant difference was identified between the groups in terms of EGFR expression (P=0.024). In total, 10 patients survived (four EGFR-positive and six EGFR-negative cases), and of the 37 deceased patients, 24 were EGFR-positive and 13 were EGFR-negative. In addition, correlations between OS and gender (P=0.021), age (P=0.018), T stage (P=0.035) and tumor location (P=0.023) were detected in the Cox proportional hazard model. Local recurrence was found to correlate with T (P=0.015) and M (P=0.026) stage, while distant metastasis was found to correlate with age (P=0.048).

## Discussion

In the present study, the expression of EGFR in a series of 47 locally advanced ESCC patients was studied. In total, 59.6% of the biopsy specimens exhibited overexpression of EGFR on immunohistochemical analysis. EGFR overexpression was revealed to correlate with the presence of lymph node metastasis and poor survival.

Previous studies have assessed the correlations between EGFR overexpression and clinicopathological features in ESCC. Firstly, certain studies have suggested that the expression of EGFR significantly correlates with depth of invasion in ESCC (14,15). The authors considered EGFR overexpression to be a predictor of T stage. However, in the current study, no correlation was observed between the expression of EGFR and T stage (P=0.593). This result may be explained by the fact that the T stage was determined by imaging and not by pathology. Secondly, the expression of EGFR in lymph node-positive groups was higher than in the negative groups (P=0.011). It must be noted that the majority of the enrolled patients were locally advanced and exhibited positive lymph nodes. Therefore, the results may correlate with the deviation of the sample size. However, other previous studies (14–17) have also demonstrated that EGFR amplification or overexpression significantly correlates with lymph node metastasis. Thirdly, the correlation between EGFR expression and the differentiation degree of ESCC remains unclear. In a study by Sunpaweravong *et al* (16), high-level protein expression of EGFR was found to correlate with well-differentiated tumors (P=0.02), while a correlation (P=0.032) was found between EGFR overexpression and poorly differentiated histology in a study by Zhang *et al* (18). However, in the present study, no significant correlation was found between the expression of EGFR and the differentiation degree of ESCC. This may be the result of a small sample size. Finally, no significant correlations were detected between the expression of EGFR and other parameters.

Previously, hyperexpression of HER-2 in the tumor has been found to correlate with ESCC progression and is significantly more common in patients developing early local relapses or distant metastases following surgery, however, this correlation has not been found in EGFR (19), as shown in the current study. This suggests that EGFR may not be a predictive factor for local relapses or distant metastases in ESCC. Although, in a study by Yamamoto *et al* (6), EGFR in the surgical group of patients was found to independently correlate with postoperative recurrence (P=0.036). In the current study, the survival rate of EGFR-positive patients appeared worse than that for EGFR-negative patients following CCRT. However, a prospective study (12) reported no correlation between EGFR expression and the OS in ESCC patients who underwent neoadjuvant chemoradiotherapy and subsequent esophagectomy. In addition, a certain study (22) found no correlation between EGFR overexpression and ESCC. In the chemotherapy group of a previous study (6), EGFR-positive patients showed an improved prognosis (P=0.022). We conclude that EGFR expression may have a predictive value in patients with ESCC treated with CCRT. However, the number of samples analyzed in the current study was small and the results require confirmation in a greater number of patients. In addition, the median follow-up time was only 15 months; therefore, the follow-up of these patients must be continued in the future. The results of a study by Gotoh *et al* (5) suggested that EGFR may aid in predicting the response of primary sites to definitive CRT in esophageal SCC, and that EGFR is not predictive of the response to concurrent CRT. With regard to the retrospective nature of the current study, inadequate information was available with regard to the patients details.

In the present study, 38 patients did not reach T4 stage and did not receive resection of the esophageal carcinoma. This was due to intolerability and unwillingness. In addition, concerning the curability of treatment for advanced localized esophageal cancer, no clear difference has previously been identified between surgery and radical CRT (1–3), and even local advanced esophageal cancer impossible to curatively resect has been reported to be cured by CRT alone in specific patients (23). In the present study, the tumor tissue of 10 patients was investigated for mutation status, but no mutations were found and the incidence of EGFR mutations in patients with ESCC was extremely low. Therefore, the correlation between the presence of EGFR mutations and clinicopathological features and outcomes was not studied following CCRT.

In conclusion, EGFR overexpression may be observed as a potentially useful biomarker, clinically; however, further larger and more homogeneous prospective studies are required to demonstrate the predictive value of EGFR for ESCC patients who have received CCRT.

## Figures and Tables

**Table I:** Patient characteristics.

	Patients	
	
Characteristic	n	%
Age, years		
<60	20	42.6
≥60	27	57.4
Gender		
Male	30	63.8
Female	17	36.2
Location		
Cervical	4	8.5
Upper	25	53.2
Middle	14	29.8
Lower	4	8.5
Histological grade		
G1	13	27.7
G2	21	44.6
G3	13	27.7
T stage		
≤T2^a^	8	17
T3	30	63.8
T4	9	19.2
N stage		
N0	10	21.3
N1	37	78.7
M stage		
M0	41	87.2
M1a	6	12.8
TNM stage		
II	14	29.8
III	33	70.2

a Since the T stage was determined by imaging and not by pathology, T1 to T2 were combined.

G1, well-differentiated; G2, moderately differentiated; G3, poorly differentiated; T, depth of invasion; N, lymph node metastasis; M, distant metastasis.

**Table II:** Results of the logistic regression analysis between the expression of EGFR and clinicopathological features.

Features	B	SE	Wald	df	P-value	Exp (B)
Gender	−2.130	1.372	2.411	1	0.120	0.119
Age, years	−0.180	1.214	0.022	1	0.882	0.836
G	0.648	1.175	0.304	1	0.582	1.911
T	−1.092	2.045	0.285	1	0.593	0.335
N	−6.445	2.520	6.541	1	0.011^a^	0.002
M	−4.121	2.115	3.798	1	0.051	0.016
Location	−1.531	1.521	1.014	1	0.314	0.216

a P<0.05, indicating a statistically significant difference.

EGFR, epidermal growth factor receptor; G, pathological differentiation; T, depth of invasion; N, lymph node metastasis; M, distant metastasis.
